# Comparing estimated protein excretion rate and spot urinary protein-creatinine ratio in assessing urinary protein excretion in patients with kidney disease in China: a single center study

**DOI:** 10.3389/fmed.2025.1517019

**Published:** 2025-03-06

**Authors:** Yu Jia, Lingling Zhao, Fang Wang, Jin Shang, Zhanzheng Zhao

**Affiliations:** ^1^Nephrology Laboratory, The First Affiliated Hospital of Zhengzhou University, Zhengzhou, China; ^2^Department of Nephrology, The First Affiliated Hospital of Zhengzhou University, Zhengzhou, China; ^3^State Key Laboratory of Medical Genomics, National Research Center for Translational Medicine at Shanghai, Shanghai Institute of Hematology, Ruijin Hospital Affiliated to Shanghai Jiao Tong University School of Medicine, Shanghai, China; ^4^Department of Hematology, The First Affiliated Hospital of Zhengzhou University, Zhengzhou, China; ^5^Laboratory Animal of Academy of Medical Sciences, Zhengzhou University, Zhengzhou, China

**Keywords:** kidney disease, urinary protein excretion, ePER, uPCR, 24-h urinary protein excretion

## Abstract

**Objectives:**

This study aimed to analyze the potential of the estimated protein excretion rate (ePER) as a substitute for the spot urinary protein-creatinine ratio (uPCR) in clinical reports for accurately assessing urinary protein excretion in China.

**Methods:**

We included 1721 patients in the study and compared the differences in levels, correlation, bias, methodological evaluation between uPCR, ePER, and 24-h urinary protein.

**Results:**

Significant differences (*Z* = −17.568, *p* < 0.001) were found between uPCR and 24-h urine protein levels in all cases. However, no statistically significant difference (*Z* = −0.652, *p* = 0.514) was found between ePER and 24-h urine protein. The bias analysis revealed that the negative bias rate between ePER and 24-h urine protein was −4.33%, significantly lower compared to uPCR (−30.88%). Incorporating ePER significantly boosted its sensitivity to 91.3% in this cohort. Furthermore, ePER demonstrated a higher correlation (*r* = 0.74, *p <* 0.001) and kappa consistency (*κ* = 0.802, *p* = 0.015) with 24-h urinary protein compared to uPCR (*r* = 0.71, *p <* 0.001; κ = 0.737, *p* = 0.016). However, in the >65 age group, those with estimated glomerular filtration rate (eGFR) < 30 mL/min/1.73m^2^ group and spot urinary creatinine <500 mg/L exhibited a higher ePER bias compared to uPCR.

**Conclusion:**

These findings highlight the potential of ePER as a valuable tool for accurately assessing urinary protein excretion. Nonetheless, its limitations should be considered, especially in specific patient populations.

## 1 Introduction

Proteinuria is a primary symptom of kidney disease. Evaluating proteinuria aids in diagnosis, disease monitoring, and treatment effectiveness assessment (1, 2). The “gold standard” method for the assessment of proteinuria involves quantitative determination of protein concentration in a 24-h urine collection, termed “timed urine collection.” Protein fluctuation during the day has only a slight impact on the results. However, the conventional method of quantifying protein concentration in a 24-h urine collection is time-consuming, troublesome for patients, and prone to errors such as accidental contamination, which can significantly affect its accuracy (3–5). An alternative, quick, and simple method for quantitatively evaluating urinary protein excretion may be the assessment of spot (random) urine protein or the albumin-to-creatinine ratio (uPCR/uACR). This method uses creatinine concentration in a spot urine sample as an internal control and offers a faster and simpler alternative to timed urine collection.

Literature confirms that evaluating uPCR/uACR in a single urine sample is a viable alternative to 24-h urine collection for assessing proteinuria, especially in patients with renal insufficiency (6–8). However, uPCR/uACR has limitations as it may vary according to age, sex, weight, and muscle mass (9, 10). Ix et al. developed a new equation to estimate creatinine excretion rate (eCER) based on age, sex, weight, and race (11). This equation utilizes commonly available variables and exhibits minimal bias and moderate precision, making it useful for assessing the accuracy of timed urine collection. However, weight as a predictor variable cannot be automatically applied to laboratory reports. In a separate study, Fotheringham et al. derived and validated a creatinine excretion rate (CER) estimation equation relying solely on age, sex, and race (12). The formula not only confirmed the validity of the equation but also demonstrated its superiority over uPCR and uACR in measuring protein and albumin excretion rates from timed 24-h urine collection. The formula was more convenient for clinical application, but regrettably, there is no Chinese population in the selected objects of this study. Racial and ethnic disparities represent a substantial factor influencing the evaluation of urinary protein. Research has shown that variations in urinary creatinine excretion are linked to race and ethnicity. More specifically, the uPCR may systematically underestimate 24-h proteinuria in Black and Hispanic individuals, while overestimating it in White individuals (13). Due to the large population in China and the significant number of complex cases among kidney disease patients, so it is important to verify the validity of this formula in Chinese population for clinical application.

In our study, our objective was to investigate whether ePER offers advantages compared to uPCR and to explore the potential application of ePER in clinical reports, resulting in a more accurate evaluation of urinary protein excretion in patients with kidney disease in China.

## 2 Materials and methods

### 2.1 Population and ethics

This is a prospective study utilizing historical data. This clinical study was conducted at the First Affiliated Hospital of Zhengzhou University between April 2020 and September 2020, encompassing a total of 1721 patients diagnosed with various kidney diseases. All patients during this period utilized the same batch of uPCR reagents and 24-h urine protein assay reagents, in conjunction with consistent internal quality control procedures, thereby minimizing potential systematic errors across the datasets. The inclusion criteria required morning spot urine samples collected on the same day that patients completed the 24-h urine collection while patients without kidney disease were excluded. The Ethics Review Committee of the First Affiliated Hospital of Zhengzhou University approved this study (approval ID: 2023-KY-0810) according to institutional guidelines and waived the requirement to obtain informed consent due to its non-interventional nature.

### 2.2 Collection of clinical data

We reviewed baseline demographics, clinical, and laboratory data, extracting information on age, sex, eGFR, and body mass index (BMI) from medical records. eGFR (mL/min/1.73m^2^) for males = 141 × [SCr (mg/dL)/0.9]^−0.411^ × 0.993^age^ if SCr < 0.9 mg/dL, 141 × [SCr (mg/dL)/0.9]^−1.209^ × 0.993^age^ if SCr > 0.9 mg/dL; eGFR (mL/min/1.73m^2^) for females = 144 × [SCr (mg/dL)/0.7]^−0.329^ × 0.993^age^ if SCr < 0.7 mg/dL, 144 × [SCr (mg/dL)/0.7]^−1.209^ × 0.993^age^ if SCr > 0.7 mg/dL (14).

The ePER and eAER were calculated by multiplying the uPCR and uACR by the eCER. The eCER was determined using the following formula: eCER (mg/24 h) = 1307.3 + (23.1 × age) − (0.3 × age^2) for males and 1051.1 + (5.3 × age) − (0.1 × age^2) for females (12).

### 2.3 Statistical analysis

Basic information including sex, age, diseases, BMI, eGFR, spot urine creatinine, and proteinuria between groups was compared, with continuous variables tested for normality. Categorical data were expressed as percentages, while continuous variables were described as mean ± standard deviation (SD) or median (interquartile range) depending on normality. The Chi-square test was employed for categorical data analysis, while variables with non-normal distribution were assessed using the Wilcoxon signed-rank or Mann–Whitney U test. Bias was assessed as the median difference between 24-h urine protein and uPCR or ePER. Linear regression analysis, along with Spearman’s correlation coefficient (r) (15), was conducted to explore the correlation among uPCR, ePER, uACR, eAER, and 24-h urine protein or albumin concentration. In general, the following regression coefficients were utilized to determine the degree of correlation: 0.7–1.0 denotes a strong correlation, 0.4–0.7 indicates a slight correlation, 0.2–0.4 signifies a weak correlation, and 0–0.2 suggests almost no correlation. Cohen’s kappa coefficient (*κ*) (16) was calculated to evaluate the consistency between uPCR, ePER, uACR, eAER, and 24-h urine protein or albumin. The κ coefficient was employed to define the level of agreement: < 0 suggests poor agreement, 0–0.20 denotes minimal agreement, 0.21–0.40 represents fair agreement, 0.41–0.60 indicates moderate agreement, 0.61–0.80 signifies substantial agreement, and 0.81–1.0 implies almost perfect or perfect agreement. Bias was calculated as the median difference between uPCR, ePER, uACR, eAER, and 24-h urine protein or albumin levels. The sensitivity and specificity for detecting severely increased proteinuria and albuminuria (17) were evaluated using cut-off points of 1,000 mg/24 h and 300 mg/24 h, respectively. A proteinuria threshold of 1,000 mg/24 h previously has been recommended as an indication for lower blood pressure targets (18).

All statistical analyses were performed using SPSS version 26 software (IBM Corp., Armonk, NY, United States), with statistical significance defined as *p* < 0.05.

## 3 Results

### 3.1 Baseline characteristics of participants

A population sample comprising 1721 individuals aged 9 to 93 years was included in this study. Among them, 539 were Membranous nephropathy (MN), 110 were Lupus nephritis (LN), 132 were Minimal change disease (MCD), 107 were Diabetic nephropathy (DN), 252 were IgA nephropathy (IgAN), 311 were chronic kidney disease (CKD), and 270 were classified as others. The median age of participants was 47 years. Descriptive statistics for the study cohort are presented in Table 1.

**Table 1:** Patients’ characteristics.

Variable	All (*n* = 1721)
Male, *n* (%)	1,027 (59.67)
Age, y	47 (32, 56)
Diseases, *n* (%)	
MN	539 (31.32)
LN	110 (6.39)
MCD	132 (7.67)
DN	107 (6.22)
IgAN	252 (14.64)
CKD	311 (18.07)
Others	270 (15.69)
Body mass index, kg/m^2^	25.10 (23.66, 27.34)
eGFR, mL/min/1.73m^2^	79.44 (33.19, 105.30)
Spot urine creatinine, mg/L	904.98 (576.92, 1510.18)
Proteinuria	
24-h urine protein, mg	1560.0 (401.50, 4327.75)
uPCR, mg/g	1113.84 (264.26, 3138.86)
ePER, mg/24 h	1513.51 (358.62, 4368.01)
24-h urine albumin, mg	795.90 (124.07, 2228.94)
uACR, mg/g	489.64 (73.05, 1530.32)
eAER, mg/24 h	670.81 (97.84, 2090.31)

MN, membranous nephropathy; LN, lupus nephritis; MCD, minimal change disease; DN, diabetic nephropathy; IgAN, IgA nephropathy; CKD, chronic kidney disease; eGFR, estimated glomerular filtration rate; uPCR, urine protein-to-creatinine ratio; ePER, estimated protein excretion rate; uACR, urinary albumin-creatinine ratio; eAER, estimated albumin excretion rate.

### 3.2 Bias analysis, methodological evaluation and correlation analysis of ePER and eAER

The levels of uPCR and 24-h urine protein showed significant differences (*Z* = −17.568, *p* < 0.001) across all cases in terms of urinary protein levels. However, there was no significant difference (*Z* = −0.652, *p* = 0.514) between ePER and 24-h urine protein levels. We evaluated the bias, sensitivity, specificity, consistency, and correlation of uPCR, ePER, uACR, and eAER against 24-h urine protein as the reference standard in the datasets (Table 2). Bias analysis revealed a negative bias rate of −30.88% between uPCR and 24-h urine protein, while the bias rate reduced to −4.33% between ePER and 24-h urine protein. In the methodological evaluation, although ePER (89.1%) marginally compromised the specificity of urine protein assessment compared with uPCR (92.5%), ePER (91.3%) significantly enhanced the sensitivity relative to uPCR (83.1%) in this cohort. Additionally, the *κ* coefficient of ePER (κ = 0.802, *p* = 0.015) was higher than uPCR (κ = 0.737, *p* = 0.016) in kappa consistency analysis. Similar results were found in ACR versus eAER.

**Table 2:** Bias, methodological evaluation and correlations of ePER and eAER.

	Bias (mg/day)	Bias (%)	Sensitivity (%)	Specificity (%)	κ	*r*	*p*-value
uPCR	−200.67 (−1144.02, 18.17)	−30.88 (−54.38, 2.9)	83.1	92.5	0.737	0.71	0.000
ePER	−23.28 (−371.29, 523.62)	−4.33 (−34.39, 35.40)	91.3	89.1	0.802	0.74	0.514
uACR	−83.72 (−625.76, 0.85)	−33.45 (−58.53, 1.68)	87.7	95.0	0.797	0.73	0.000
eAER	−5.0 (−233.90, 189.74)	−8.60 (−39.81, 37.21)	93.1	92.0	0.843	0.76	0.015

Bias is given as the median difference between uPCR, ePER, uACR, eAER and 24-h urine protein or albumin; The sensitivity and specificity for detecting proteinuria and albuminuria were evaluated using cut-off points of 1,000 mg/24 h and 300 mg/24 h, respectively. uPCR, urine protein-to-creatinine ratio; ePER, estimated protein excretion rate; uACR, urinary albumin-creatinine ratio; eAER, estimated albumin excretion rate; κ, kappa statistic for uPCR, ePER, uACR, eAER consistency with 24-h urine protein and albumin collection; r, correlations of uPCR, ePER, uACR, eAER and 24-h urine protein or albumin. *p*-values (vs. 24-h urine protein or albumin) < 0.05, calculated by Wilcoxon Signed Ranked Test.

### 3.3 Comparison between the improved group and the non-improved group

To assess how effectively ePER perform compared to uPCR in evaluating urinary protein excretion and their influencing factors, we divided the results into two groups: improved and non-improved (Table 3). We defined the improved group as cases where |24-h urine protein – ePER| < |24-h urine protein – uPCR|. Conversely, the non-improved group included cases where |24-h urine protein – ePER| > |24-h urine protein – uPCR|. We then compared these two groups (there were no data with the same results between uPCR and ePER in this study cohort). As indicated in Table 3, we found no significant differences in gender (χ^2^ = 0.120, *p* < 0.729) and 24-h protein values (*Z* = −0.914, *p* = 0.361) between the two groups. However, significant differences emerged in age (*Z* = −5.436, *p* < 0.001), BMI (*Z* = −3.198, *p* = 0.001), eGFR (*Z* = −12.192, *p* < 0.001), and spot urine creatinine levels (*Z* = −16.712, *p* < 0.001) between them. Similar results were observed for urinary albumin excretion (Supplementary Table 1).

**Table 3:** The comparison between the improved group and the non-improved group in the urinary protein excretion.

	The improved group (*n* = 1,144)	The non-improved group (*n* = 577)	*p*-value
Male, *n* (%)	686 (59.97)	341 (59.10)	0.729
Age, y	45 (31, 54)	50 (37, 54)	0.000
Body mass index, kg/m^2^	24.80 (23.66, 27.17)	25.40 (24.22, 27.34)	0.001
eGFR, mL/min/1.73m^2^	89.69 (55.25, 108.88)	44.85 (12.27, 93.46)	0.000
Spot urine creatinine, mg/L	1,125 (723.98, 1798.64)	622.17 (441.18, 916.29)	0.000
24-h urine protein, mg	1494.2 (390.0, 4303.75)	1674.0 (433.08, 4438.53)	0.361

eGFR, estimated glomerular filtration rate; *p*-values < 0.05, calculated by Mann-Whiney U test.

### 3.4 Comparison of levels and correlation of ePER with 24-h urine protein in different groups

We compared uPCR and ePER with 24-h urinary protein levels across various groups, as presented in Table 4. The results showed that in males (*Z* = −18.875, *p* < 0.001) and females (*Z* = −3.392, *p* = 0.001), < 18 years old (*Z* = −4.505, *p* < 0.001), BMI < 24 kg/m^2^ (*Z* = −11.636, *p* < 0.001) and 24–28 kg/m^2^ (*Z* = −10.495, *p* < 0.001), eGFR 30–59 mL/min/1.73 m^2^ (*Z* = −5.669, *p* < 0.001) and 60–89 mL/min/1.73 m^2^ (*Z* = −9.646, *p* < 0.001) groups, uPCR and 24-h urine protein were statistically significant. While no statistically significant differences were found between ePER and 24-h urinary protein levels in these groups. Interestingly, there were statistically significant difference between ePER and 24-h urinary protein in age > 65 years (*Z* = −6.534, *p* < 0.001), BMI > 28 kg/m^2^ (*Z* = −2.397, *p* = 0.017), eGFR <30 mL/min/1.73 m^2^ (*Z* = −11.130, *p* < 0.001) groups. But there was no significant difference between uPCR and 24-h urinary protein levels. The correlation among uPCR, ePER, and 24-h urine protein levels was meticulously analyzed in each group (Table 4). In the male group, uPCR exhibited a slight correlation with 24-h urinary protein (*r* = 0.694, *p* < 0.001), as did the eGFR 60-89 mL/min/1.73 m^2^ group (*r* = 0.699, *p* < 0.001). However, ePER demonstrated a strong correlation with 24-h urinary protein levels in both groups, with correlation coefficients of 0.709 (*p* < 0.001) and 0.763 (*p* < 0.001), respectively. Furthermore, the correlation between both uPCR and ePER with 24-h urinary protein was strong, except for the age > 65 years and eGFR <30 mL/min/1.73 m^2^ group, where only a slight correlation was observed. Additionally, the magnitude of the correlation coefficient between ePER and 24-h urinary protein consistently exceeded that of uPCR in almost all groups, except for the >65 years group. We performed the same analysis for urinary albumin excretion (Supplementary Table 2).

**Table 4:** Comparison levels and correlation of ePER with 24-h urine protein in different groups.

Group	*n*	24-h urine protein, mg	uPCR, mg/g	ePER, mg/g	*p*^a^ value	*p*^b^ value	*r_1_*	*r_2_*
Gender								
Male	1,027	2075.52 (483.00, 5311.0)	1242.85 (284.15, 3203.49)	2060.12 (488.83, 5348.15)	0.000	NS	0.694	0.709
Female	694	1078.35 (323.94, 2970.0)	962.34 (236.81, 2951.93)	1209.01 (255.43, 3085.04)	0.001	NS	0.794	0.80
Age, y								
<18	82	986.04 (198.75, 3847.0)	719.84 (167.86, 3121.12)	988.96 (245.86, 3869.09)	0.000	NS	0.765	0.792
18–49	911	1445.0 (408.0, 4032.48)	905.56 (235.73, 2630.08)	1290.12 (335.52, 3995.74)	0.000	0.000	0.747	0.776
50–65	541	1872.0 (438.0, 4934.52)	1339.39 (304.69, 3537.56)	1832.04 (389.81, 4758.28)	0.000	0.012	0.729	0.754
>65	187	1725.0 (415.80, 4625.60)	2062.67 (420.95, 4942.36)	2328.92 (524.45, 5615.06)	NS	0.000	0.656	0.639
Body mass index, kg/m^2^								
<24	349	2304.54 (525.0, 5526.63)	1218.29 (248.77, 3440.67)	2046.60 (413.39, 5920.20)	0.000	NS	0.768	0.772
24–28	643	1530.0 (396.0, 4129.60)	1127.10 (284.84, 3105.05)	1522.66 (402.12, 4203.74)	0.000	NS	0.780	0.802
>28	216	1188.50 (397.68, 3439.50)	1275.56 (364.11, 3618.58)	1400.96 (381.36, 3983.19)	NS	0.017	0.835	0.843
eGFR, mL/min/1.73m^2^								
<30	370	2645.86 (1320.0, 5581.50)	2663.79 (1492.06, 5293.95)	3754.04 (1906.30, 7289.72)	NS	0.000	0.665	0.667
30–59	227	1530.0 (440.0, 4625.60)	1237.6 (358.38, 3392.09)	1615.96 (467.44, 4821.61)	0.000	NS	0.828	0.864
60–89	339	1180.0 (338.0, 4121.0)	821.96 (204.00, 2596.75)	1198.71 (287.77, 3751.61)	0.000	NS	0.699	0.763
>90	671	1120.50 (310.35, 3803.80)	576.52 (179.36, 2081.68)	839.13 (225.87, 2987.03)	0.000	0.000	0.749	0.792
Spot urine creatinine, mg/L								
<500	307	1768.0 (670.95, 5382.0)	2320.5 (904.56, 5920.74)	2688.72 (1067.78, 7930.20)	0.000	0.000	0.744	0.761
500–1,000	659	1917.70 (474.60, 4170.0)	1423.39 (371.54, 3264.0)	1936.75 (462.53, 4572.10)	0.000	0.000	0.830	0.838
1,000–1,500	321	1233.0 (300.0, 4002.0)	736.67 (165.11, 2176.48)	1035.67 (233.45, 3279.37)	0.000	0.000	0.825	0.839
1,500–2000	182	1003.95 (336.0, 4230.0)	552.26 (158.90, 2184.26)	683.18 (210.88, 3125.36)	0.000	0.000	0.844	0.901
>2000	253	1008.0 (240.0, 4358.0)	419.71 (90.93, 2066.44)	623.91 (130.98, 2930.18)	0.000	0.000	0.737	0.771

eGFR, estimated glomerular filtration rate; uPCR, urine protein-to-creatinine ratio; ePER, estimated protein excretion rate; *p*^a^ < 0.05, Wilcoxon signed-rank test of uPCR and 24-h urine protein; *p*^b^ < 0.05, Wilcoxon signed-rank test of ePER and 24-h urine protein; r_1_, correlation of uPCR and 24-h urine protein; r_2_, correlation of ePER and 24-h urine protein. NS, no significance.

### 3.5 Comparing bias and methodological evaluation of ePER with 24-h urine protein in different groups

We conducted a bias analysis and methodological evaluation of outcomes obtained from uPCR and ePER compared with 24-h urine protein measurements across various study cohorts (Supplementary Table 3). Based on bias analysis, ePER demonstrated a lower bias rate than uPCR in most groups. For instance, the uPCR exhibited a bias rate of −40.14% for males and −14.02% for females, while the ePER showed a reduced bias rate of merely −0.47% and − 9.35%, respectively. However, the bias rates of ePER compared to uPCR were increased in age > 65 years (26.50% vs. 5.63%), BMI > 28 kg/m^2^ (6.72% vs. 1.32%), eGFR <30 mL/min/1.73 m^2^ (32.34% vs. 0.90%), and spot urine creatinine <500 mg/L (45.92% vs. −15.91%) groups.

Regarding methodological evaluation, the utilization of ePER led to a slight reduction in specificity but significantly enhanced sensitivity in urine protein assessment across nearly all cohorts. The *κ* coefficients of ePER showed higher values across most groups compared to uPCR, indicating substantial or nearly perfect consistency with 24-h urinary protein measurements. However, when considering individuals with an eGFR <30 mL/min/1.73 m^2^, the κ coefficient indicated only moderate agreement between uPCR (κ = 0.587, *p* = 0.056) or ePER (κ = 0.580, *p* = 0.059) and the gold standard for assessing urinary protein levels. We performed the same analysis for urinary albumin excretion (Supplementary Table 4).

## 4 Discussion

Quantifying proteinuria is crucial for clinically assessing patients with kidney diseases due to its strong correlation with renal prognosis (19–22). While the gold standard remains 24-h urine protein measurement, random uPCR is commonly used due to the challenges associated with collecting complete 24-h urine samples. The existing literature has consistently demonstrated a strong correlation between uPCR and 24-h urinary protein levels (23, 24). However, some studies have shown that the two are only moderately correlated and consistent (25). Studies have also highlighted clinically unacceptable deviations between 24-h urinary protein levels and uPCR values (26). Therefore, a new method using spot urine protein and creatinine values that is able to minimize under or over estimation is still warranted. In our study, we demonstrated the potential of ePER as a valuable tool for accurately assessing urinary protein excretion. This research validates the effectiveness of ePER in optimizing uPCR for evaluating urinary protein excretion in a Chinese population. Despite certain limitations, ePER effectively mitigated discrepancies between uPCR and 24-h urine protein levels.

In this study, we compared the uPCR using the newly calculated ePER based on a formula (12). Our results confirm that this formula can also optimize uPCR results in most cases in the Chinese population. The application of ePER not only significantly reduced the discrepancy between uPCR and 24-h urine protein levels but also improved relevance and consistency. Methodological evaluation showed that the utilization of ePER led to a slight decrease in specificity while significantly enhancing the sensitivity of urine protein assessment across nearly all cohorts. These findings align with those of a previous study (12). Based on the data from this study, the ePER derived from an age- and sex-based formula appears to mitigate the confounding effect of sex, allowing both males and females to effectively minimize the disparity between uPCR and 24-h urinary protein levels. Additionally, no statistically significant difference was found in the 24-h protein content between the improved and non-improved groups, suggesting that changes in urinary protein levels did not affect the optimization effect of ePER on uPCR.

In this study, the use of ePER greatly improved the uPCR results in the group under 65 years of age, and the difference between uPCR and 24-h urinary protein was greatly reduced, especially in adolescent patients under 18 years of age. However, despite using a formula derived from age and sex optimization to calculate ePER, the impact of age remains partially unaccounted for in the findings of this study. In this study, it is evident that ePER does not completely mitigate the influence of age and is not applicable to patients aged >65 years, in contrast to the findings of Fotheringham et al. (12). These contradictory results on the one hand may arise from disparities in population selection, with the equation derivation and validation cohorts of previous study (12) containing only a small number of participants older than 70 years and none older than 80 years, and on the other hand may be related to disease characteristics, treatment approaches, and sample size.

For BMI group comparison, uPCR results were more affected at low BMI, and the bias rate from 24-h urine protein was the largest, up to −46.12%. However, the effect of this factor was well reduced by the use of ePER, and the bias rate was less than −10%. Although ePER did not play an optimization role in BMI > 28 kg/m^2^, the bias rate was only 6.72%. Unfortunately, a previous study (12) did not consider grouping BMI, nor did it provide analytical discussions on these subgroups.

In a comparative study of urinary protein excretion in the eGFR group, uPCR had the best correlation with 24-h urinary protein in the eGFR 30–59 mL/min/1.73m^2^ group and a moderate correlation in the eGFR <30 mL/min/1.73m^2^ group. This is consistent with the findings of Ahmed et al.[21], moderate correlation (*r* = 0.535) was seen in patients with advanced renal failure (eGFR <15 mL/min/1.73m^2^). However, it is worth noting that the bias rates of uPCR and 24-h urine protein were positively correlated with eGFR values. The bias rate of the eGFR <30 mL/min/1.73m^2^ group was only 0.9%, which was much lower than that of other eGFR groups. The use of ePER not only increased the correlation with 24-h urine protein, but also significantly reduced the bias rate in these groups, especially for patients with eGFR 30–89 mL/min/1.73m^2^. But unfortunately, when eGFR was <30 mL/min/1.73 m^2^, the application of ePER did not improve uPCR results, which is consistent with results previously reported by other researchers (12).

Additionally, when spot urine creatinine levels <500 mg/L, ePER performs less effectively compared to uPCR. However, when spot urine creatinine >500 mg/L, ePER played a different degree of optimization, especially when spot urine creatinine was 500–1,000 mg/L. In this study, the reasons why ePER was not optimized were related to old age, obesity, low eGFR, and low urinary creatinine point. However, the main factor may be related to urinary creatinine excretion rate, because more than half of the patients in the no-improvement group had spot urine creatinine level < 500 mg/L. And other indicators also more or less affected the urinary creatinine excretion rate. Previous investigations have shown a correlation between age and urinary creatinine excretion rate, with a decline observed as age increases (27, 28). Taylor et al. found that urinary creatinine excretion rate increased with BMI (29), and Negri et al. reached the same conclusion for both men and women (30). Moreover, previous studies have suggested that individuals with lower eGFR tend to have lower urinary creatinine excretion rates (8, 30, 31). To validate our study, we recruited 244 additional patients with positive urine protein and tested their urine protein and creatinine in morning urine and 24-h urine (As some studies are still ongoing, complete data are not yet available). We conducted a preliminary analysis of the data from this batch of patients and found that ePER (6.46%) had the lowest bias rate and was more favorable than the 24-h uPCR (−11.49%). Through this data analysis, we were able to validate the research findings presented in this study.

In this study, the implementation of ePER effectively reduced the disparity between uPCR and 24-h urinary protein levels. However, certain limitations were identified. The current study demonstrated that ePER performed better than uPCR in assessing urinary protein excretion in most cases, laying the groundwork for its future practical application of in China. However, uPCR showed certain advantages in specific subgroups, with limited optimization by ePER, displaying a superior bias rate, correlation, and consistency. This indicates that a combined approach using both ePER and uPCR may be more suitable in clinical practice at present.

Unfortunately, 24-h urinary creatinine data were unavailable for this analysis due to its non-routine use in renal function assessment, a limitation of the study. In future practice, we will prioritize incorporating 24-h urine creatinine into our protocols and compare uPCR, ePER, and 24-h urine protein excretion using an adequate database to enhance renal function evaluations. This effort is designed to identify a simple and reliable protein excretion test that can potentially eliminate the need for 24-h urine collection. We believe ePER would become a powerful tool for kidney diseases screening.

In conclusion, this study validates the effectiveness of ePER in optimizing uPCR for evaluating urinary protein excretion in a Chinese population. Despite some limitations, ePER effectively reduced disparities between uPCR and 24-h urine protein levels. Advanced age, obesity, reduced eGFR, and abnormal urinary creatinine excretion were identified as significant determinants impacting the efficacy of ePER improvement. This study suggests that the combination of ePER and uPCR holds promise for practical applications, pending further validation using clinical data.

## Data Availability

The original contributions presented in the study are included in the article/Supplementary material, further inquiries can be directed to the corresponding authors.
