# Supplementary material for: Comparing estimated protein excretion rate and spot urinary protein-creatinine ratio in assessing urinary protein excretion in patients with kidney disease in China: a single center study
Source: Front Med (Lausanne). 2025 Mar 6;12:1517019. doi: 10.3389/fmed.2025.1517019 (PMC11922839; doi:10.3389/fmed.2025.1517019)
Supplement: Supplementary file 1 [file Table_1.docx]

Supplementary Material

# Supplementary Tables

**
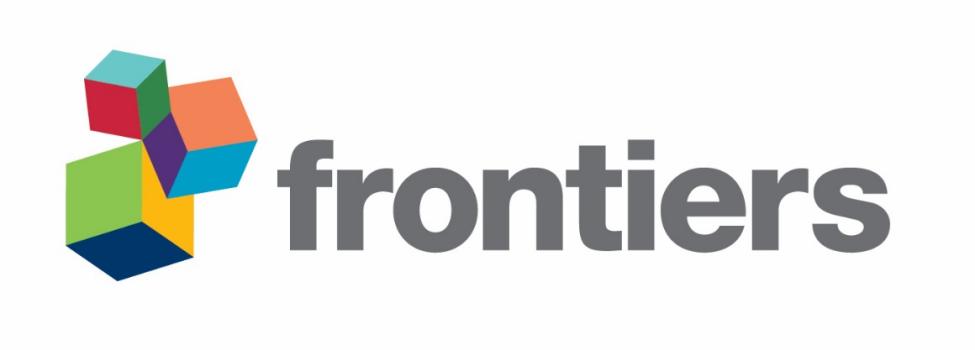
**

**Supplementary Table 1** The comparison between the improved group and the non-improved group in the urinary albumin excretion.

|  | The improved group(n = 1141) | The non-improved group(n = 580) | *p*-value |
| --- | --- | --- | --- |
| Male, n(%) | 693(60.74) | 334(57.59) | 0.208 |
| Age, y | 45(31, 54) | 50(36, 60) | 0.001 |
| Body mass index, kg/m^2^ | 24.80(23.39, 26.76) | 25.71(24.22, 27.34) | 0.000 |
| eGFR, mL/min/1.73m^2^ | 88.96(52.68,108.62) | 48.73(13.23, 94.84) | 0.000 |
| Spot urine creatinine, mg/L | 1097.29(690.05, 1742.08) | 644.80(452.49, 970.02) | 0.000 |
| 24-h urine albumin, mg | 803.16(128.31, 2340.11) | 754.18(113.44, 2073.49) | 0.090 |

Note: The improved group: |24-hour urine albumin protein － eAER| < |24-hour albumin protein － uACR|; The non-improved group: |24-hour albumin protein － eAER| > |24-hour albumin protein － uACR|.

Abbreviations: eGFR, estimated glomerular fifiltration rate; *p*-values < 0.05, calculated by Mann-Whiney U test.

**Supplementary Table 2** Comparison levels and correlation of eAER with 24-h urine albumin in different groups.

| Group | n | 24-h urine albumin, mg | uACR, mg/g | eAER, mg/24h | *p^a^* value | *p^b^* value | *r_1_* | *r_2_* |
| --- | --- | --- | --- | --- | --- | --- | --- | --- |
| **Gender** |  |  |  |  |  |  |  |  |
| Male | 1027 | 1011.20(172.48, 2835.56) | 590.01(95.03, 1597.34) | 975.82(160.50, 2623.34) | 0.000 | NS | 0.723 | 0.734 |
| Female | 694 | 428.08(88.76, 1537.12) | 374.94(55.53, 1391.76) | 405.45(59.02, 1503.52) | 0.000 | 0.001 | 0.802 | 0.809 |
| **Age, y** | | | | | | | | |
| <18 | 82 | 429.09(33.01, 2237.58) | 346.57(18.84, 1307.93) | 465.71(30.01, 1935.02) | 0.000 | NS | 0.737 | 0.760 |
| 18-49 | 911 | 770.88(141.65, 2070.0) | 412.27(74.82, 1275.37) | 612.11(101.02, 1894.42) | 0.000 | 0.000 | 0.741 | 0.764 |
| 50-65 | 541 | 911.20(123.13, 2432.47) | 593.82(76.83, 1732.69) | 791.14(100.29, 2343.42) | 0.000 | NS | 0.733 | 0.762 |
| >65 | 187 | 758.99(97.02, 2391.38) | 895.39(113.80, 2496.04) | 1003.20(122.42, 2908.78) | NS | 0.000 | 0.780 | 0.788 |
| **Body mass index, kg/m^2^** | | | | | | | | |
| <24 | 349 | 1057.92(189.75, 2895.03) | 538.47(83.29, 1658.02) | 925.42(135.76, 2812.69) | 0.000 | NS | 0.759 | 0.763 |
| 24-28 | 643 | 795.96(111.45, 2136.96) | 501.82(77.53, 1536.80) | 722.44(103.74, 2020.38) | 0.000 | NS | 0.751 | 0.772 |
| >28 | 216 | 546.12(121.61, 1691.61) | 508.77(109.12, 1688.0) | 509.88(111.58, 1848.10) | NS | NS | 0.822 | 0.834 |
| **eGFR, mL/min/1.73m^2^** | | | | | | | | |
| <30 | 370 | 1195.62(421.98, 2689.59) | 1159(464.94, 2452.47) | 1600.21(627.34, 3292.51) | NS | 0.000 | 0.747 | 0.749 |
| 30-59 | 227 | 815.34(142.32, 2581.20) | 595.58(94.58, 1747.87) | 809.33(116.46, 2471.64) | 0.000 | NS | 0.819 | 0.857 |
| 60-89 | 339 | 551.16(95.80, 2133.43) | 384.14(47.69, 1364.11) | 560.20(69.17, 1925.66) | 0.000 | 0.004 | 0.716 | 0.782 |
| >90 | 671 | 567.36(94.83, 1986.88) | 300.56(34.43, 1141.06) | 404.95(46.15, 1649.01) | 0.000 | 0.000 | 0.727 | 0.762 |
| **Spot urine creatinine, mg/L** | | | | | | | | |
| <500 | 307 | 911.2(176.65, 2618.89) | 1021.36(225.0, 2943.72) | 1262.58(260.84, 3810.03) | 0.000 | 0.000 | 0.815 | 0.841 |
| 500-1000 | 659 | 938.56(160.20, 2225.88) | 678.58(112.43, 1634.08) | 887.16(142.45, 2118.53) | 0.000 | NS | 0.816 | 0.824 |
| 1000-1500 | 321 | 584.40(99.20, 2055.72) | 367.90(46.48, 1139.29) | 491.12(67.47, 1719.46) | 0.000 | 0.000 | 0.839 | 0.851 |
| 1500-2000 | 182 | 505.78(105.54, 2203.56) | 264.82(47.58, 1202.80) | 371.77(59.63, 1787.66) | 0.000 | 0.000 | 0.845 | 0.897 |
| >2000 | 253 | 505.44(38.66, 1988.01) | 185.76(13.53, 988.00) | 282.06(20.57, 1516.98) | 0.000 | 0.000 | 0.737 | 0.772 |

Abbreviations: eGFR, estimated glomerular fifiltration rate; uACR, urinary albumin-to-creatinine ratio; eAER, estimated albumin excretion rate; *p*^a^<0.05, Wilcoxon signed-rank test of ACR and 24-h urine albumin; *p*^b^<0.05, Wilcoxon signed-rank test of eAER and 24-h urine albumin; *r*_1_, correlation of uACR and 24-h urine albumin; *r*_2_, correlation of eAER and 24-h urine albumin. NS, no significance.

**Supplementary Table 3** Bias analysis and methodological evaluation of ePER in the cohort.

| Group | n | Bias (mg/day) | Bias(%) | Sensitivity(%) | Specificity(%) | κ |
| --- | --- | --- | --- | --- | --- | --- |
| **Gender** |  |  |  |  |  |  |
| Male |  |  |  |  |  |  |
| uPCR | 1027 | -448.39(-1731.88, -75.32) | -40.14(-60.26, -13.38) | 83.2 | 91.4 | 0.661 |
| ePER |  | -4.16(-453.68, 825.52) | -0.47(-32.21, 43.23) | 93.0 | 83.1 | 0.754 |
| Female |  |  |  |  |  |  |
| uPCR | 694 | -61.75(-367.06, 225.16) | -15.02(-41.27, 21.70) | 87.6 | 91.2 | 0.787 |
| ePER |  | -43.09(-294.05, 297.64) | -9.35(-37.05, 25.65) | 90.1 | 90.6 | 0.807 |
| **Age, y** |  |  |  |  |  |  |
| <18 |  |  |  |  |  |  |
| uPCR | 82 | -148.50(-1041.52, -9.69) | -28.95(-53.13, -0.95) | 90.2 | 97.6 | 0.878 |
| ePER |  | 13.34 (-201.53, 441.72) | 5.05(-31.11, 38.87) | 92.7 | 92.7 | 0.854 |
| 18-49 |  |  |  |  |  |  |
| uPCR | 911 | -336.0(-1333.50, -59.27) | -40.74(-60.04, -14.01) | 77.0 | 93.2 | 0.680 |
| ePER |  | -78.68(-548.62, 287.04) | -15.54(-39.13, 22.40) | 88.6 | 89.6 | 0.776 |
| 50-65 |  |  |  |  |  |  |
| uPCR | 541 | -147.15(-1179.06, 91.55) | -25.01(-48.88, 7.97) | 87.7 | 91.9 | 0.779 |
| ePER |  | 18.69(-326.80, 731.46) | 2.85(-32.91, 46.99) | 93.4 | 88.5 | 0.820 |
| >65 |  |  |  |  |  |  |
| uPCR | 187 | 30.38(-276.78, 709.17) | 5.63(-24.35, 40.56) | 94.8 | 87.3 | 0.828 |
| ePER |  | 219.45(-28.78, 1402.96) | 26.50(-4.33, 71.08) | 97.4 | 85.9 | 0.850 |
| **Body mass index, kg/m^2^** | | | | | | |
| < 24 |  |  |  |  |  |  |
| uPCR | 349 | -521.56 (-1985.55, -98.25) | -46.12 (-63.83, -20.18) | 77.9 | 92.7 | 0.656 |
| ePER |  | -62.26 (-597.66, 855.28) | -9.35 (-38.70, 34.07) | 89.8 | 88.6 | 0.772 |
| 24-28 |  |  |  |  |  |  |
| uPCR | 643 | -175.50(-979.98, 31.58) | -28.11(-51.57, 4.76) | 84.4 | 93.9 | 0.765 |
| ePER |  | -14.65(-324.17, 528.28) | -2.70(-54.80, 3.05) | 64.9 | 89.4 | 0.512 |
| > 28 |  |  |  |  |  |  |
| uPCR | 216 | 7.06(-325.81, 450.29) | 1.32(-32.50, 41.18) | 92.2 | 84.0 | 0.766 |
| ePER |  | 25.86(-184.13, 594.28) | 6.72(-26.52, 48.82) | 93.1 | 82.0 | 0.756 |
| **eGFR, mL/min/1.73m^2^** | | | | | | |
| < 30 |  |  |  |  |  |  |
| uPCR | 370 | 13.07(-714.08, 758.63) | 0.90(-26.40, 40.39) | 95.0 | 59.4 | 0.587 |
| ePER |  | 665.32(22.17, 2649.86) | 32.34(1.23, 88.46) | 98.7 | 49.3 | 0.580 |
| 30-59 |  |  |  |  |  |  |
| uPCR | 227 | -200.67(-1140.61, 86.93) | -22.13(-46.96, 13.90) | 88.6 | 92.6 | 0.803 |
| ePER |  | 11.96(-290.55, 445.13) | 3.17(-23.49, 45.92) | 95.5 | 91.6 | 0.873 |
| 60-89 |  |  |  |  |  |  |
| uPCR | 339 | -212.0(-1147.44, -24.0) | -35.38(-54.11, -4.83) | 79.8 | 94.9 | 0.736 |
| ePER |  | -38.24(-375.78, 251.74) | -8.70(-34.71, 24.01) | 91.8 | 91.7 | 0.834 |
| > 90 |  |  |  |  |  |  |
| uPCR | 671 | -325.3(-1472.59, -65.94) | -44.50(-62.98, -20.71) | 72.3 | 97.2 | 0.689 |
| ePER |  | -106.59(-655.66, 50.55) | -24.08(-45.13, 9.10) | 83.3 | 94.8 | 0.777 |
| **Spot urine creatinine, mg/L** | | | | | | |
| < 500 |  |  |  |  |  |  |
| uPCR | 307 | 211.50(-147.15, 11720.0) | -15.91(-12.67, 62.09) | 96.0 | 71.6 | 0.709 |
| ePER |  | 657.84(91.97, 2707.41) | 45.92(8.85, 100.20) | 99.0 | 63.3 | 0.676 |
| 500-1000 |  |  |  |  |  |  |
| uPCR | 659 | -203.51(-1008.15, 19.33) | -22.44(-42.58, 3.26) | 87.0 | 94.5 | 0.784 |
| ePER |  | 48.56(-230.40, 738.99) | 6.31(-20.40, 43.64) | 92.9 | 89.8 | 0.822 |
| 1000-1500 |  |  |  |  |  |  |
| uPCR | 321 | -353.09(-1562.24, -64.08) | -42.40(-56.45, -17.21) | 75.1 | 94.4 | 0.680 |
| ePER |  | -70.30(-513.08, 80.71) | -15.04(-35.78, 12.86) | 86.4 | 93.8 | 0.794 |
| 1500-2000 |  |  |  |  |  |  |
| uPCR | 182 | -427.11(-1744.68, -118.05) | -53.0(-64.53, -34.70) | 71.4 | 100.0 | 0.714 |
| ePER |  | -212.94(-1071.55, -40.23) | -33.19(-48.20, -9.23) | 90.1 | 97.8 | 0.879 |
| > 2000 |  |  |  |  |  |  |
| uPCR | 253 | -440.87(-2254.64, -115.10) | -62.13(-71.52, -42.84) | 69.3 | 99.2 | 0.684 |
| ePER |  | -207.83(-1381.69, -60.87) | -40.78(-57.53, -19.29) | 81.9 | 98.4 | 0.802 |

Note: Bias is given as the median difference between uPCR, ePER, and 24-h urine protein; The sensitivity and specificity for detecting proteinuria was evaluated using cut-off points of 1000 mg/24h.

Abbreviations: uPCR, urine protein-to-creatinine ratio; ePER, estimated protein excretion rate; κ, kappa statistic for uPCR, ePER consistency with 24-h urine protein.

**Supplementary Table 4** Bias analysis and methodological evaluation of eAER in the cohort.

| Group | n | Bias (mg/day) | Bias(%) | Sensitivity(%) | Specificity(%) | κ |
| --- | --- | --- | --- | --- | --- | --- |
| **Gender** |  |  |  |  |  |  |
| Male |  |  |  |  |  |  |
| ACR | 1027 | -215.47(-953.44, -4.78) | -44.33(-63.33, 13.66) | 86.1 | 95.5 | 0.759 |
| eAER |  | -2.44(-282.15, 324.23) | -5.40(-38.07, 42.20) | 93.6 | 90.7 | 0.830 |
| Female |  |  |  |  |  |  |
| ACR | 694 | -11.31(-46.10, 23.14) | -17.84(-46.10, 23.14) | 90.5 | 94.4 | 0.843 |
| eAER |  | -7.29(-190.90, 77.72) | -11.71(-43.36, 29.13) | 92.3 | 93.4 | 0.854 |
| **Age, y** |  |  |  |  |  |  |
| < 18 |  |  |  |  |  |  |
| ACR | 82 | -29.76(-734.71, -0.15) | -31.81(-55.28, -1.56) | 95.3 | 97.4 | 0.926 |
| eAER |  | -0.43(-235.08, 96.27) | -2.76(-33.04, 47.93) | 97.7 | 94.7 | 0.926 |
| 18-49 |  |  |  |  |  |  |
| ACR | 911 | -140.64(-720.58, -2.48) | -43.41(-62.75, -11.62) | 84.1 | 95.3 | 0.751 |
| eAER |  | -24.60(-292.27, 93.66) | -17.16(-43.48, 25.31) | 91.1 | 92.1 | 0.815 |
| 50-65 |  |  |  |  |  |  |
| ACR | 541 | -59.38(-639.84, 16.80) | -29.28(-56.16, 12.38) | 89.7 | 94.8 | 0.819 |
| eAER |  | -1.37(-221.54, 241.95) | -2.23(-38.52, 44.27) | 94.3 | 91.6 | 0.855 |
| > 65 |  |  |  |  |  |  |
| ACR | 187 | -1.97(-170.99, 186.19) | -2.60(-29.96, 35.16) | 97.5 | 94.2 | 0.919 |
| eAER |  | 47.50(-14.28, 630.49) | 47.50(-14.28, 630.49) | 98.3 | 91.3 | 0.907 |
| **Body mass index, kg/m^2^** | | | | | | |
| < 24 |  |  |  |  |  |  |
| ACR | 349 | -272.19(-1039.12, -15.38) | -48.20(-65.75, -21.71) | 72.2 | 97.0 | 0.687 |
| eAER |  | -23.75(-314.88, 264.53) | -13.33(-42.93, 30.50) | 87.2 | 92.3 | 0.794 |
| 24-28 |  |  |  |  |  |  |
| ACR | 643 | -69.90(-548.0, 3.05) | -30.0(-54.01, 6.30) | 88.7 | 96.2 | 0.822 |
| eAER |  | -1.66(-219.48, 208.71) | -5.18(-37.14, 46.69) | 94.9 | 93.6 | 0.880 |
| > 28 |  |  |  |  |  |  |
| ACR | 216 | -1.77(-213.78, 151.75) | -5.84(-38.82, 36.26) | 89.2 | 89.5 | 0.780 |
| eAER |  | 0.23(-173.27, 256.89) | 0.46(-33.32, 50.36) | 90.8 | 88.4 | 0.788 |
| **eGFR, mL/min/1.73m^2^** | | | | | | |
| < 30 |  |  |  |  |  |  |
| ACR | 370 | -20.71(-376.18, 270.81) | -6.19(-29.65, 40.74) | 96.3 | 82.7 | 0.797 |
| eAER |  | 216.17(-28.86, 1139.40) | 29.82(-6.62, 84.85) | 98.6 | 73.3 | 0.782 |
| 30-59 |  |  |  |  |  |  |
| ACR | 227 | -61.42(-617.92, 8.59) | -28.48(-49.36, 4.70) | 91.0 | 95.2 | 0.842 |
| eAER |  | -3.30(-180.05, 162.38) | -3.22(-31.73, 39.30) | 95.8 | 92.8 | 0.886 |
| 60-89 |  |  |  |  |  |  |
| ACR | 339 | -83.46(-637.82, -0.70) | -40.43(-60.27, -2.17) | 84.1 | 96.2 | 0.773 |
| eAER |  | -11.27(-272.60, 80.56) | -11.60(-41.13, 26.73) | 89.9 | 95.4 | 0.836 |
| > 90 |  |  |  |  |  |  |
| ACR | 671 | -143.52(-800.32, -2.66) | -48.34(-66.15, -19.24) | 81.6 | 97.0 | 0.756 |
| eAER |  | -40.96(-397.27, 2.98) | -27.57(-51.26, 11.36) | 89.3 | 95.2 | 0.829 |
| **Spot urine creatinine, mg/L** | | | | | | |
| < 500 |  |  |  |  |  |  |
| ACR | 307 | 31.78(-72.45, 569.58) | 16.98(-23.48, 61.07) | 96.6 | 82.0 | 0.809 |
| eAER |  | 206.62(-0.57, 1150.06) | 36.02(-1.51, 97.72) | 98.6 | 78.0 | 0.805 |
| 500-1000 |  |  |  |  |  |  |
| ACR | 659 | -88.08(-547.95, 1.71) | -26.22(-48.39, 0.98) | 92.2 | 98.6 | 0.872 |
| eAER |  | -0.38(-169.26, 306.50) | -0.62(-30.01, 43.47) | 95.8 | 93.8 | 0.889 |
| 1000-1500 |  |  |  |  |  |  |
| ACR | 321 | -184.31(-868.52, -2.19) | -44.82(-60.46, -17.43) | 80.3 | 94.9 | 0.709 |
| eAER |  | -23.32(-306.88, 30.42) | -17.91(-42.96, 16.53) | 89.2 | 92.4 | 0.797 |
| 1500-2000 |  |  |  |  |  |  |
| ACR | 182 | -215.89(-1008.04, -36.69) | -55.30(-67.19, -33.14) | 83.0 | 97.4 | 0.781 |
| eAER |  | -87.16(-574.42, -1.96) | -34.45(-50.85, -8.51) | 87.7 | 97.4 | 0.834 |
| > 2000 |  |  |  |  |  |  |
| ACR | 253 | -202.59(-1083.14, -6.28) | -61.75(-73.54, -40.83) | 74.3 | 98.2 | 0.705 |
| eAER |  | -102.35(-751.35, -1.33) | -40.41(-59.20, -13.07) | 86.4 | 97.3 | 0.826 |

Note: Bias is given as the median difference between uACR, eAER and 24-h urine albumin; The sensitivity and specificity for detecting albuminuria was evaluated using cut-off points of 300 mg/24h.

Abbreviations: uACR, urinary albumin-to-creatinine ratio; eAER, estimated albumin excretion rate; κ, kappa statistic for uACR, eAER consistency with 24-h urine albumin.
